# 

**Published:** 1894-01

**Authors:** 


**The Central Texas Medical Society** will hold its 30th quarterly session in Waco, January 9th inst. Annual election of officers and banquet. Also the question will be canvassed whether the Society shall be "migratory as to its meetings." Lady friends of delegates and invited guests are included in the invitations to the banquet. A most interesting program has been prepared. Some excellent papers will be read and discussed, and all who contemplate attending may expect a good time and be assured of a warm reception. Dr. W. H. Wilkes of Waco, is president.

---

**Surgeon Jas. Gassaway, M. H. S.,** under orders of the Surgeon-General U. S. M. H. S., during the month of December (ult.), inspected all the quarantine stations on the Gulf coast.

